# Spatiotemporal organization of cilia drives multiscale mucus swirls in model human bronchial epithelium

**DOI:** 10.1038/s41598-018-20882-4

**Published:** 2018-02-05

**Authors:** Mustapha-Kamel Khelloufi, Etienne Loiseau, Marc Jaeger, Nicolas Molinari, Pascal Chanez, Delphine Gras, Annie Viallat

**Affiliations:** 1 Aix Marseille Univ, CNRS, CINAM, Marseille, France; 2 Aix Marseille Univ, CNRS, Centrale Marseille, M2P2, Marseille, France; 3 Institut Montpelliérain Alexander Grothendieck, CNRS, Univ, Montpellier, France; 4 Department of Statistics, University of Montpellier Hospitals, Montpellier, France; 5 Aix Marseille Univ, Inserm, INRA, C2VN, Marseille, France

## Abstract

Mucociliary clearance is a biomechanical mechanism of airway protection. It consists of the active transport along the bronchial tree of the mucus, a fluid propelled by the coordinated beating of a myriad of cilia on the epithelial surface of the respiratory tract. The physics of mucus transport is poorly understood because it involves complex phenomena such as long-range hydrodynamic interactions, active collective ciliary motion, and the complex rheology of mucus. We propose a quantitative physical analysis of the ciliary activity and mucus transport on a large panel of human bronchial cultures from control subjects, patients with asthma and chronic obstructive pulmonary disease obtained from endobronchial biopsies. Here we report on the existence of multiple ciliary domains with sizes ranging from the tens of a micron to the centimeter, where ciliary beats present a circular orientational order. These domains are associated with circular mucus flow patterns, whose size scales with the average cilia density. In these domains, we find that the radial increase of the ciliated cell density coupled with the increase in the orientational order of ciliary beats result in a net local force proportional to the mucus velocity. We propose a phenomenological physical model that supports our results.

## Introduction

**Figure 1:**
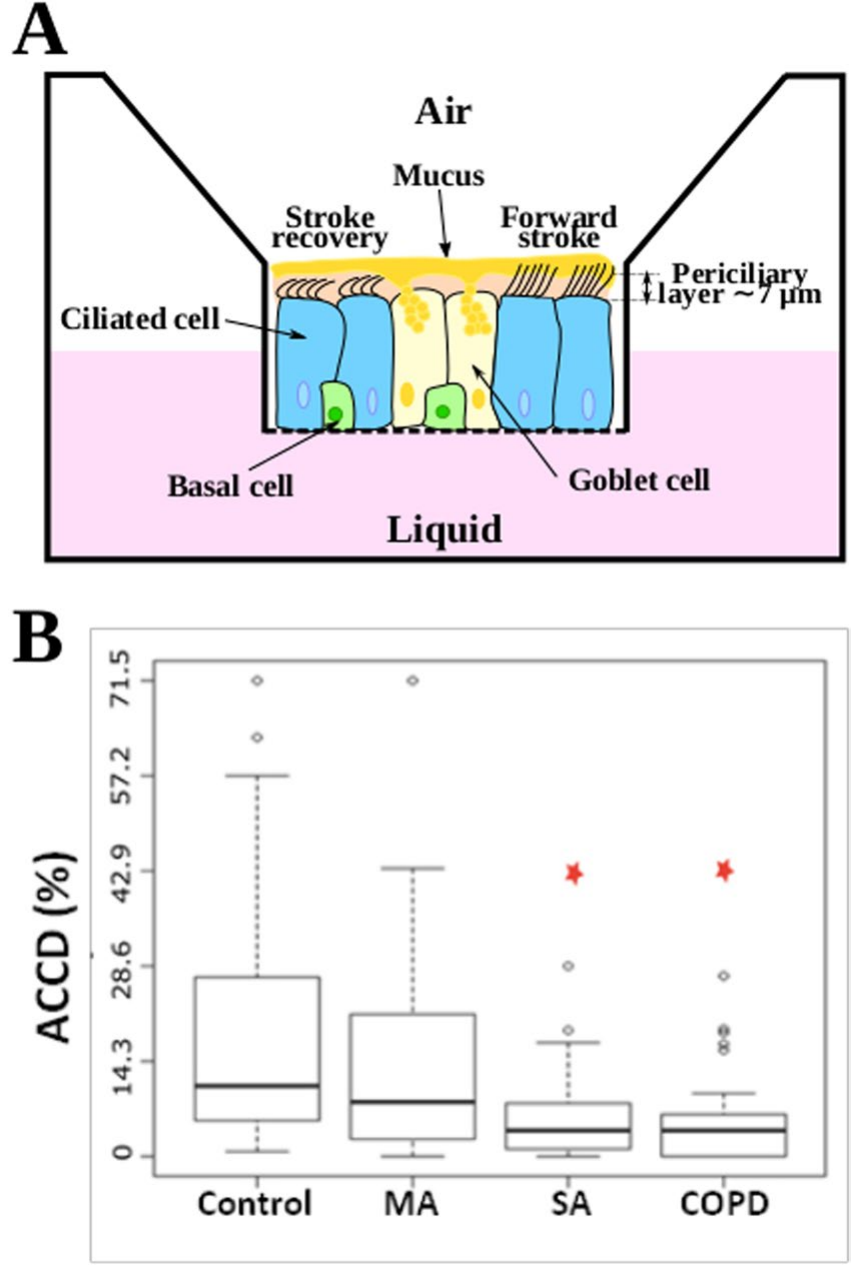
Air Liquid Interface (ALI) bronchial epithelium. **(A)** Schematic of the ALI chamber: the basal side of the differentiated epithelium is in contact with the culture medium through a porous membrane and the apical side points towards the air. The epithelium has a pseudo stratified structure made of basal cells, goblet cells which produce the mucus and ciliated cells. During the forward stroke cilia tips poke in the mucus layer while their recovery motion takes place in the periciliary layer. **(B)** Average active ciliated cell density quantified for control cultures (n = 17), mild asthma (MA, n = 7), severe asthma (SA, n = 18) and chronic obstructive pulmonary disease (COPD, n = 7). Results are expressed as median with 10 and 90 percentiles.

The continuous increase of chronic respiratory diseases worldwide, such as severe asthma, chronic obstructive pulmonary disease (COPD) and cystic fibrosis, represent a major threat for the quality of life. The pathophysiology associated to severe asthma and COPD results from an abnormal bronchial epithelium^1^ leading to a sharp increase in susceptibility to infection and inflammation^2^. The bronchial epithelium is the first barrier to protect the respiratory tract via an innate mechanism called mucociliary clearance. It consists in the active transport of a sticky fluid, the mucus, via a myriad of cilia along the epithelial surface of the airways. The mucus traps inhaled pathogens and the protective role of the mucociliary clearance relies on the ability of cilia to self-organize and coordinate their beating in order to transport the mucus along the bronchial tree till its elimination by swallowing or expectoration. From a biophysical point of view, the bronchial epithelium is an active system which involves two main key players. First, ATP-driven microscopic cilia. Located at the apical surface of epithelial ciliated cells and immersed in a swollen grafted polyanionic gel called periciliary layer (PCL)^3^, they beat asymmetrically at their own frequency, direction and phase with their tip poking in the mucus during the forward stroke (see Fig. 1A)^4,5^. Second, the mucus layer, whose complex shear-thinning properties govern its flow on top of the ciliary layer. The mucus behaves like a cohesive gel in absence of external mechanical stress and like a viscous fluid under stress^6^. Despite a large corpus of clinical and biological studies, the physical mechanisms of long-range transport of mucus remain poorly understood due to the intrinsic complexity of the mucociliary system. Indeed, the force propelling the mucus is exerted by the displacement of cilia tips in the mucus layer and depends on the patterns of individual ciliary beats, on the coordination of their phase and direction^7^ and on the nonlinear viscoelastic response of the mucus. The transport of mucus also depends on the boundary conditions, specifically on the friction between the fluid PCL and the top mucus layer, resulting in a strongly coupled system. Furthermore, the system is responsive. Ciliary beat frequencies (CBF) were found to change with the viscosity of mucus^8^, suggesting that cilia might act as motile mechanosensitive organelles able to adapt their CBF with the drag force they experience in the mucus layer. Cilia are also able to transiently change their beat directions, as observed in the brain ventricles of mice^9^. *In-vivo* and *ex-vivo* explorations of the spatiotemporal coordination of ciliary beats and its coupling to mucociliary clearance has been little experimentally studied because suitable methods like inhalation of radiolabeled markers^10^ to assess mucus velocity or methods directly using *ex-vivo* preparation of trachea are invasive. Though providing valuable information, mucociliary clearance rates measured in animal models are not fully relevant for humans, especially in mice models^11–15^. The development of well-differentiated human airway cultures at the air–liquid interface (ALI) allows overcoming previous limitations^16^. This culture model retains the mixed mucus-secretory and ciliated surface phenotype expressed *in vivo*^17^. Seminal works on cystic fibrosis^18–21^ reported that mucus could be spontaneously transported as a rigid disc in a circular pattern over the whole culture chamber for more than one week. The associated ciliary activity was however not investigated. It has been recently investigated in the ventricular system of mice brain, where highly organized directional patterns of cilia modules were discovered, leading to spatiotemporal modulated complex flow patterns of the cerebrospinal fluid, including swirls^9^. The spatiotemporal organization of ciliary activity and its interplay with mucus transport in the mucociliary clearance are still to be understood in health and disease. in particular, dyskinesia and abnormal cilia beat patterns have already been reported on bronchial brushings in severe asthma^22^. Here, we propose a quantitative physical analysis of ciliary activity and mucus transport on a large panel of human bronchial ALI cultures from control subjects, patients with mild asthma, severe asthma and COPD obtained from endobronchial biopsies. We report on the existence of multiple ciliary domains, whose sizes ranges from tens of microns to centimeters, where ciliary beats present a circular orientational order. These domains are associated with a circular mucus flow patterns, whose size scales with the average cilia density. We quantify the high degree of organization of ciliary activity on these domains and propose a phenomenological model which links mucus velocity to ciliary organization. Finally, we discuss our findings in the context of severe asthma and COPD.

## Results

We first controlled, using confocal microscopy, that ALI cultures exhibit the pseudo-stratified structure (see Fig. S1A) typical of bronchial epithelium, with basal cells, goblet cells, and ciliated cells (see the schematic Fig. 1A). The observed average ciliary length of 6.8 *μ*m is in agreement with values reported in the literature^23^. For patients suffering of severe asthma, epithelial defects such as loosely connected adjacent cells were often observed (Fig. S1B). We quantified the ciliary activity of ALI cultures from 17 control subjects, 7 patients with mild asthma, 18 patients with severe asthma and 7 patients with COPD.

### Average active ciliated cell density

The average active ciliated-cell density, *ν*, was quantified by measuring the fraction of the epithelial surface covered by beating cilia. We found that *ν* ranges from 0 to 0.70 with a large variability among different donors and an average value of 0.16 for controls. No significant change of *ν* is observed after elimination of the mucus layer at the surface of the epithelium. These values are in agreement with those previously reported on ALI cultures^24,25^, but lower than those observed in the trachea, wherein the percentage of ciliated cell is about 50%^26^. *ν* is not altered in mild asthma (*ν* = 0.142, non significant) but strongly decreases to 0.055 (p = 0.0032) and 0.0395 (p = 0.0168) in severe asthma and COPD respectively (Fig. 1B). This strong decrease supports previous studies performed on epithelial cells collected from endobronchial biopsies and observed in solution^22^. It also supports that severe asthma and COPD are epithelial diseases^1^, associated with morphological abnormalities of the bronchial epithelium.

### Frequency and orientational order of Ciliary beats

**Figure 2:**
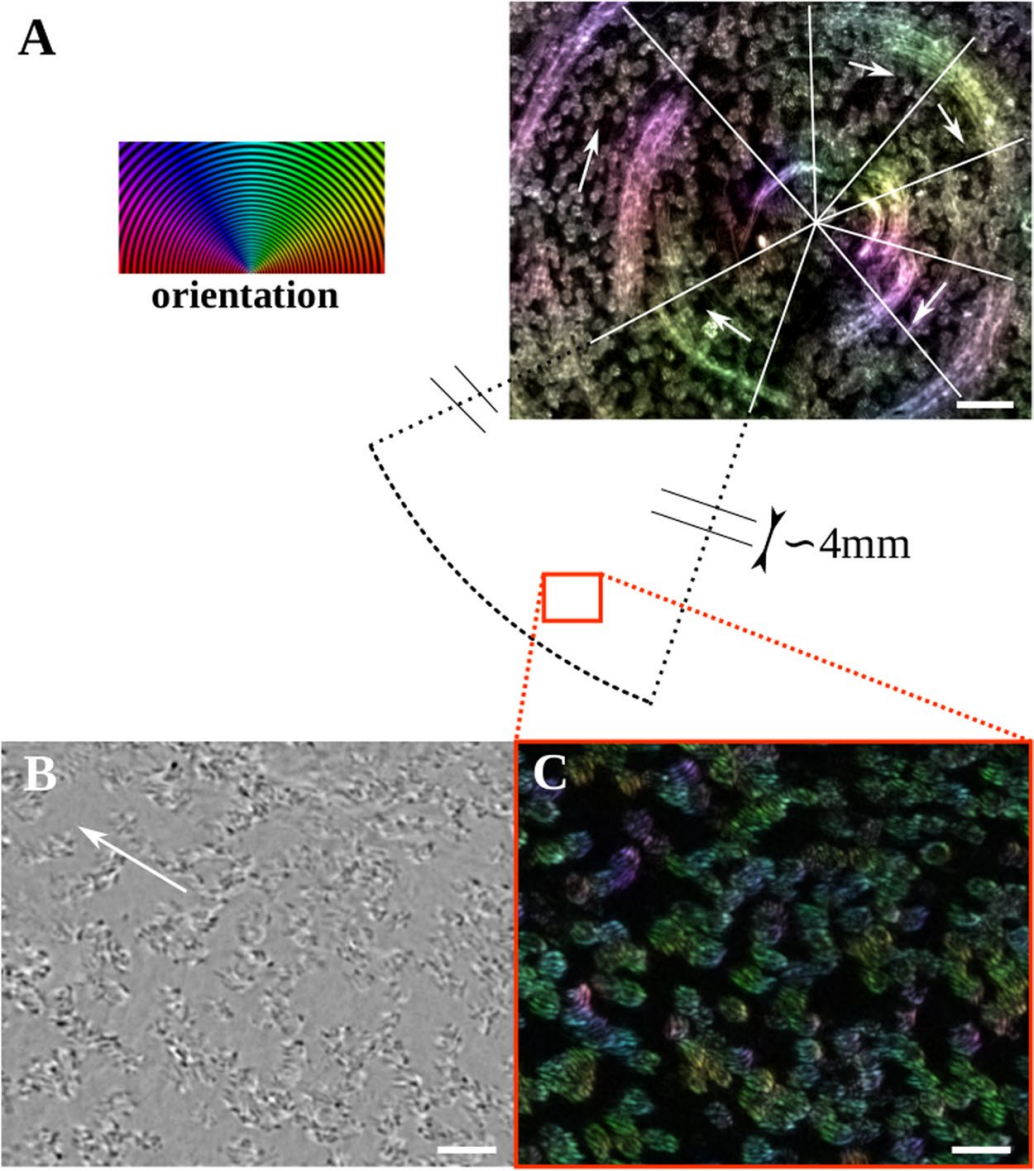
Cilia orientation map in a swirl. **(A)** The image is a standard deviation projection over 500 images acquired at 50 fps of a the central part of a domain where ciliary beats have a circular orientational order (mild asthma culture). The spots are the tufts of cilia and the trails are due to dead cells/fragments embedded in the mucus. The color codes for the orientation of both mucus flow and ciliary beats. The white lines demarcate the main areas with a preferential orientation indicated by the arrows. On average there is a correlation between ciliary beat orientations and mucus flow. Scale bar, 50 *μ*m. On the left, orientation scale. **(B** and **C)** Zoom on the cilia. Bright field image in (**B**), the white arrow indicates the direction of mucus transport. Corresponding standard deviation projection in (**C**). Scale bars, 20 *μ*m.

The average ciliary beat frequency (CBF) measured on control subject is 18.3 Hz with a dispersion of ±3.2. A typical frequency map is shown on Fig. S2 for the ciliary domain of Fig. 2B and C and Movie S1. More details on CBF in health and diseases are given in SI 1 and Fig. S2. Steric interactions between neighboring cilia impose a directional order at the scale of one ciliated cell or adjacent ciliated cells. A typical example for an isolated group of neighboring cells is shown in Movie S2, where two main beat directions are observed. Surprisingly, at a larger scale and up to the scale of the whole culture chamber, we almost systematically observed the existence of spatial domains on which ciliary beats present an striking circular orientational order. The radius of these domains ranges between 10 microns (a few cells) to the whole culture chamber (R = 6 mm). Several swirls can be found within a culture chamber, randomly distributed in the central or peripheral zones of the cell culture. (see movie S3). A typical example of an orientation map of ciliary beats is shown in Fig. 2A on an epithelium with *ν* equal to 0.2, where the circular domain spans the whole culture chamber (R = 6 mm). The tangential orientational order of the ciliary beats, illustrated on a disc in the central zone is visible in Fig. 2A. The orientation map of ciliary beats at a peripheral zone (Fig. 2B and Movie S1) of the circular domain is shown in Fig. 2C. Although ciliated cells may be distant from one another and cilia do not form a dense mat, an orientational order of ciliary beats is clearly visible. This suggests that additional mechanisms to steric interactions are involved in the coordination of beat direction. It should be noted that not all cilia are beating in the same direction (see cilia in purple, for example). We will now show that these circular orientational patterns of ciliary beats generate multiscale flows of the fluid located at the epitelial surface.

### Rotational surface fluid flows

**Figure 3:**
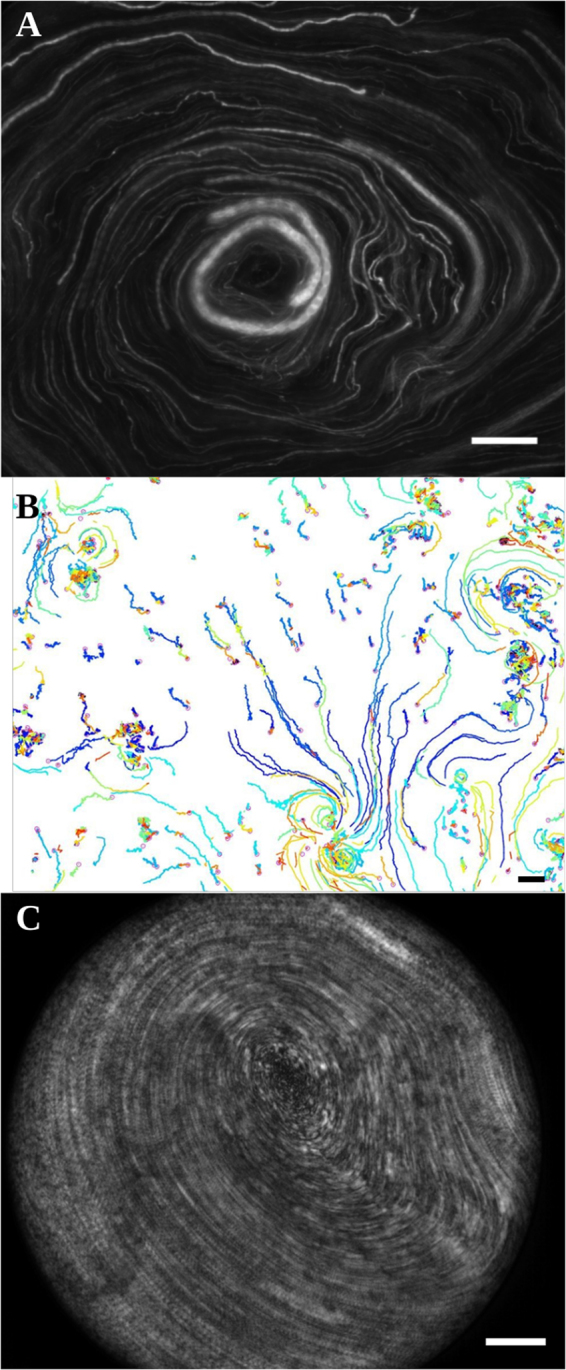
Cilia driven transport. **(A)** Transport of 1-*μ*m fluorescent beads at the apical surface of a washed epithelium. The image is a standard deviation projection over 50 images. Scale bar, 200 *μ*m. **(B)** Local transport. Trajectories are obtained by tracking 1-*μ*m beads. Scale bar, 20 *μ*m. **(C)** Rotational transport of mucus spanning the whole chamber (same sample than in **A**). The image is a standard deviation projection over 50 images. Scale bar, 200 *μ*m.

To study the ability of cilia to generate local flows, we performed an apical washing to remove the native mucus and replaced it by a dilute solution of 1-*μ*m beads in PBS. Typical bead trajectories are shown in Fig. 3A and B and movie S4. In areas lacking of ciliated cells the beads remain immobile, while elsewhere many beads present a net motion over hundreds of micrometers. Two sort of trajectories are illustrated in Fig. 3B: a linear or pseudo-linear bead motion associated with a linear spatial repartition of cilia, and, strikingly, a circular motion on domains of radii ranging from 10 *μ*m (a few ciliated cells) to hundreds of microns depending on the culture chamber. Such a transport over several hundreds of microns reveals that on these circular trajectories the local force that applies on every bead is purely tangential of the whole cellular culture. Several clockwise and counterclockwise swirls can coexist in a single chamber, together with linear flows indicating that these circular domains are independent from one another and from the geometry of the chamber. These swirls are observed at the surface of epithelial domains on which ciliary beats present a circular orientational order, thus showing that these swirls are directly generated by the self-organization of ciliary beats. Indeed, the local forces controlling the micron-size displacement of the latex beads must be remarkably organized at the macroscopic scale of the bead trajectories to ensure their circular displacement. This point will be detailed thereafter.

It should be noted that before performing an apical washing, the samples where latex beads rotate on radii of several hundreds of microns, exhibited a macroscopic rotational motion of native mucus spanning the whole chamber (see Fig. 3C and movie S3). Such a motion has been reported by Matsui *et al*.^18^. Similarly to latex bead transport, mucus was observed to rotate clockwise or counterclockwise with an equal probability, thus revealing a phenomenon of stochastic origin In many cases the angular velocity of mucus is constant (see Fig. S3), thus showing that it behaves like a rigid body. Interestingly, the mucus velocity at the edge of the chamber is of the order of 200 *μm*.*s*^−1^, similar to the range of physiological bronchial mucus velocities^14,27,28^.

### The average density of cilia sets the size of the swirls

**Figure 4:**
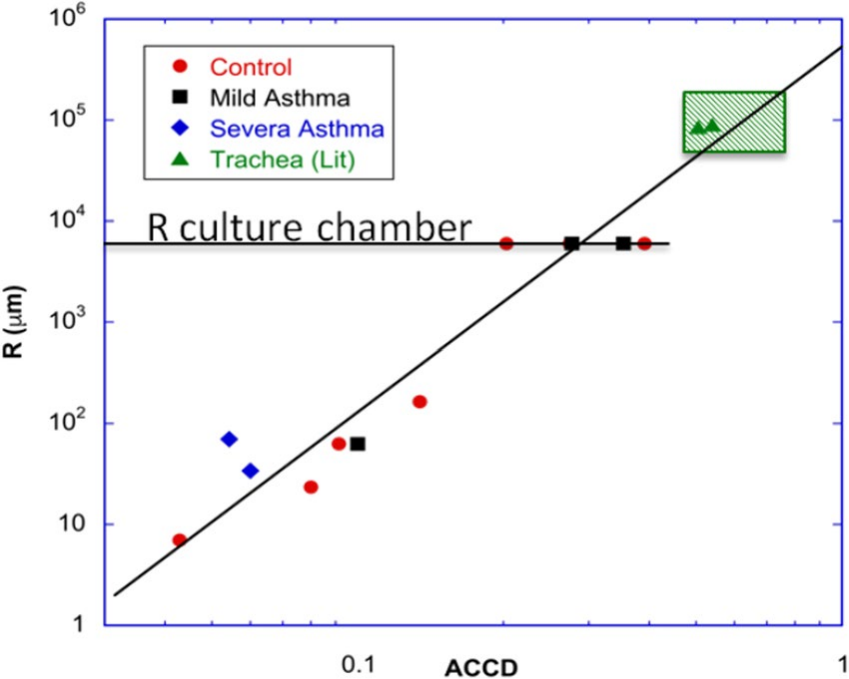
The size of swirls scales with *ν*. The radius of the swirls is plotted versus the average *ν* for control (red circles), mild asthma (black squares) and severe asthma (blue diamonds) ALI cultures. The data for the trachea (green triangles) are from the literature^26,35^. The size of the swirls scales with *ν* over 3 orders of magnitude, from R ~ 10 *μ*m up to the radius of the ALI culture (R = 6 mm). Interestingly, the data for the trachea fall on the same curve.

The size of the largest circular domains of bead/mucus transport in each culture chamber scales, via a power law, with *ν* over three orders of magnitude (see Fig. 4). For circular domains spanning the whole chamber (see Fig. 3C and Movie S5), *ν* is in the range 20–30%, indicating that a dense ciliated epithelium is not necessary to drive the mucus flow at the centimeter scale. In addition, physiological data reported in the literature on mucus transport in the trachea fall on the same curve. This master curve points out that the average ciliated cell density directly governs the size of epithelial surface on which a directional flow transport is observed, or in other words, the distance over which a surface fluid (beads in a Newtonian fluid or mucus) can be transported along the epithelium. This curve may be one key to understand mucus congestion. In particular, this curve shows that low *ν* values observed for patients with severe asthma and COPD displayed in Fig. 1B might be the main cause of altered mucociliary clearance.

### Experimental analysis of the local forces exerted on swirls

**Figure 5:**
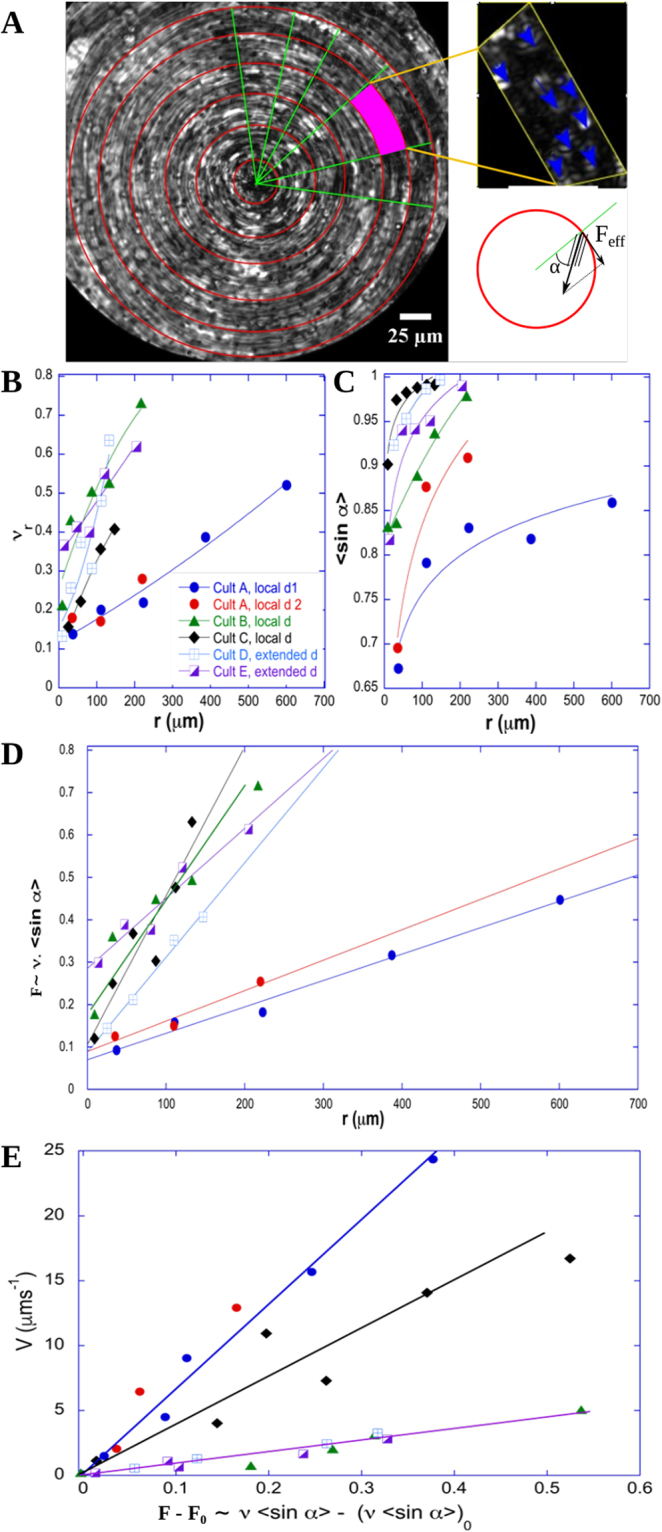
Driving force on 6 swirls. **(A)** The size and the center of a swirl are determined on an image, which results from the projection of the maximum intensity of the acquired movie. 1-*μ*m fluorescent beads are added on the mucus to facilitate the visualization. The standard deviation projection of the movie results in an image on which we can quantify *ν* and the ciliary beat directions on annuli. *α* is the angle between the ciliary beat direction of a ciliary tuft and the radius passing through the tuft. **(B)** The evolution of *ν* measured on an annulus at distance r from the center of the swirl, *ν*_*r*_, is plotted versus r for 6 different swirls. **(C)** The evolution of Sin *α* averaged on an annulus at distance r from the center of the swirl, <*Sin α*>, is plotted versus r for the swirls analyzed in (**B**) **(D)** The propulsive force, proportional to the product of *ν*_*r*_ with <*Sin α*> increases radially in an affine way. **(E)** The curvilinear mucus velocity increases linearly with the propulsive force.

The circular transport of mucus at a constant angular velocity on a disk requires a fine regulation over the entire disk, in terms of direction and magnitude, of the forces that locally propel the mucus. Indeed, along a diameter, the local forces should have the same tangential direction and an opposite direction on either side of the center. The magnitude of local forces must also vary along the radius to drive a transport at a constant angular velocity, that is with a curvilinear velocity which increases linearly with the distance from the center of the swirl. In this section we quantify how such a fine regulation is provided by the ciliated epithelium. We analyse, within a swirl, the spatial and dynamic self-organization of the cilia underneath the mucus for 6 domains from 5 different culture chambers. We performed the experiments on commercial cultures from healthy human samples (MucilAir from Epithelix, Switzerland). Most commercial Epithelix cultures are characterized by a high *ν* (more than 65%) and thus, exhibit mucus rotation over the whole culture chamber (the radius of the chamber is 3.25 mm). We studied two such cultures. Three other cultures were provided by Epithelix at a younger stage, with epithelial cells still being differentiated. On reception, the number of ciliated cells in the culture was small and *ν* was therefore low. After elimination of mucus and 1-*μ*m beads addition, no significant bead motion was observed. After a few days, upon increase of *ν*, large rotational flow domains were observed at the epithelial surface, where patches of mucus rotated at constant angular velocity during more than 24 hours (Fig. S3 and Movie S3). The existence of local rotational flows and epithelial domains with an orientational order of ciliary beats, both on cultures prepared in our laboratory and on commercial ones, clearly show the general and robust nature of these phenomena. To decipher the physical parameters that determine the mucus propelling force on a rotational flow domain, we consider that each active cilium exerts a force on the surface fluid when the cilium tip penetrates the fluid. This force has the direction of the ciliary beat and its useful component is the tangential one, which circularly propels the surface fluid (see Fig. 5A). At first approximation we assume that the force exerted on the surface fluid by an area element of the epithelium is proportional to *ν* on this area element. By decomposing the rotational domain into concentric annuli, the force per unit area on an annulus located at a distance r from the center of the domain is therefore proportional to *ν*_*r*_ <*sin α*>_*r*_, with *α* the angle between a beat direction and the radial direction of the circular domain, *ν*_*r*_ and <*sin α*>_*r*_ are *ν* and the average of sin *α* on the considered annulus.

We quantified both *ν*_*r*_ and < *sinα* >_*r*_ for different values of r on each rotational domain. Their respective radial evolutions are plotted in Fig. 5B and C. Both the local ciliated density and the average tangential component of ciliary beats increase with the distance to the center of the domain. Moreover, the product *ν*_*r*_ <*sinα*>_*r*_, which is proportional to the mucus driving force, increases linearly with r on each localized domains (A to C) and on the central part (r < 200 *μ*m) of the two entire chamber-spanning domains D and E (see Fig. 5D). This remarkable radial increase of the driving force ensures a stable circular movement of the mucus. These results show that there is a fine spatial organization of the epithelium in terms of ciliary beat direction and cilia density. The adequate local cilia density could be regulated during the initial process of differentiation or by direct transdifferentiation without proliferation among epithelial cells^29^. This transdifferentiation might be triggered by mechanotransduction due to the resistive mechanical force exerted by the periciliary layer and by the viscoelastic mucus on beating cilia. Another way to spatially regulate cilia density is the re-arrangement of the ciliated cells within the epithelium. Indeed we observed over hours the displacement of the cells with an average drift of 22 *μ*m/h. This re-arrangement could lead in changes in cell orientations and planar cell polarity, which might drive changes in beating direction^7^. In addition, the radial increase in orientational order might result from steric constraints between neighboring cilia due to the radial increase of *ν*.

Interestingly, the force per unit area does not vanish at the center of a domain. Its value is expected to be directly linked to the resistive friction exerted by the periciliary layer on the surface mucus layer. Finally, as both the local force per unit area and the tangential velocity of the surface fluid vary linearly with r, they also vary linearly with each other as shown in Fig. 5E. Consequently, the mucus is transported at constant angular velocity. It is therefore not radially sheared in these rotational domains, thus limiting viscous dissipation. This is energetically favorable but requires a high degree of self-organization of spatial cilia density and temporal ciliary beats. The physical interpretation of this linear relationship is not straightforward. In order to better understand the origin of this minimal force that has to be applied to generate a surface-fluid flow and the linear velocity-force relationship, we developed a semi-quantitative phenomenological model that we now detail.

### Phenomenological model

**Figure 6:**
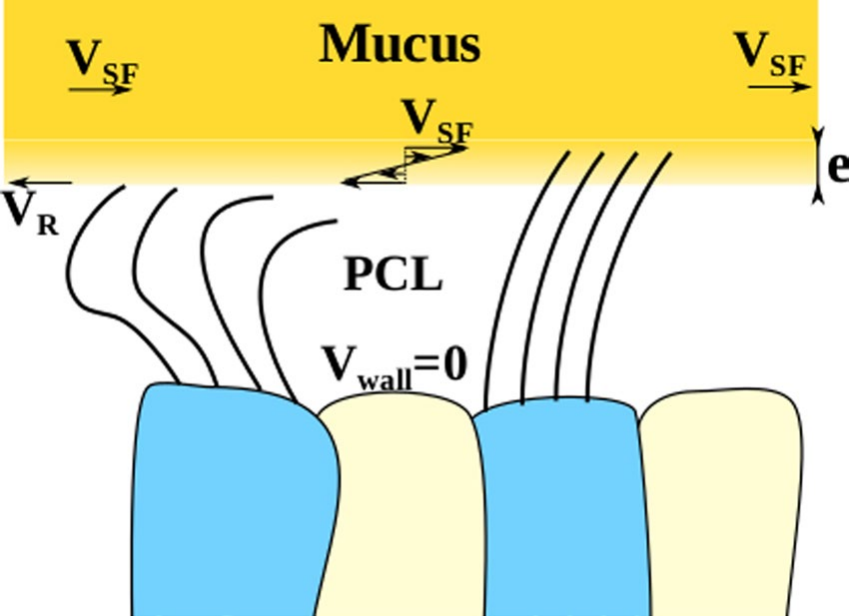
Schematic of the mucociliary coupled system. Geometry of the mucociliary system considered for the phenomenological model. V_*R*_ is the recovery ciliary velocity, V_*SF*_ is the surface fluid velocity, e is the thickness of the sheared layer.

The linear relationship between velocity and force results from two opposite actions on the surface mucus layer, the propulsion exerted by cilia and the resistive friction exerted by the periciliary layer. Here we propose a model that describes the mucociliary system as a periciliary layer with cilia tufts separated by empty zones, where cilia beat near a no-slip wall. Above, the mucus is considered as a yield-stress fluid (Fig. 6). We hypothesize that the yield-stress is reached only when cilia tips penetrate into mucus. Everywhere else mucus behaves like a rigid body and has a constant velocity V_*SF*_. Within a circular domain, the propulsive force per unit area exerted in an annulus at a distance r from the center writes as:

1 $$\frac{{\phi }\,{n}_{c}\,{f}_{c}}{{A}_{cc}}\,{\nu }_{r} < sin\,\alpha { > }_{r},$$

where *φ* is the time fraction during which the propulsion occurs, *n*_*c*_ is the average number of cilia per ciliated cell, *A*_*cc*_ is the area of a ciliated cell and *f*_*c*_ is the force exerted by one cilium to the surface mucus layer. *f*_*c*_ results from the motion of the cilium tip in mucus and is expected to be proportional to the CBF and the fluid viscosity (see SI 2). The resistive friction force arises during the recovery of the cilia to their initial position. They drag the periciliary fluid backward in the ciliated zones and in the neighboring non-ciliated zones, whereas the top of the periciliary layer is dragged forward by mucus. This results in a shear layer in the upper part of the periciliary layer as shown in Fig. 6. The stress in this layer in an annulus at distance r from the center of the circular domain is ${v}_{r}(1-\phi ){\eta }_{pc}\frac{({V}_{SF}(r)+{V}_{R})}{e}$, in ciliated zones and $(1-{v}_{r}){\eta }_{pc}\frac{({V}_{SF}(r)+{V}_{R})}{e}$ in neighboring non-ciliated zones, where e is the thickness of the sheared layer, *η*_*pc*_ is the viscosity of the periciliary fluid, and *V*_*R*_ is the velocity of the recovering cilia tips. The shear in the lower part of the periciliary layer is lower (small variation in speed on a large fluid thickness). The total stress is $\tau =(1-\phi {v}_{r}){\eta }_{pc}\frac{({V}_{SF}(r)+{V}_{R})}{e}\approx {\eta }_{pc}\frac{({V}_{SF}(r)+{V}_{R})}{e}$ since *φν*_*r*_ ≪ 1. The balance between propulsive and resistive force yields an affine relation of the force with the surface fluid velocity, in agreement with experimental results:

2 $$\frac{{\phi }\,{n}_{c}\,{f}_{c}}{{A}_{cc}}\,{\nu }_{r}\, < sin\,\alpha { > }_{r}={\eta }_{pc}\,\frac{({V}_{SF}(r)+{V}_{R})}{e}.$$

According to Eq. 2, the constant friction term observed at r = 0 is directly linked to the backward cilia velocity. Therefore, the slope variations observed in Fig. 5E may be due to the viscosity of the periciliary layer, the number of cilia per ciliated cell or the ciliary beat pattern motion. The slope of the variations of *V*_*SF*_ versus *ν*_*r*_<*sinα*>_*r*_, $\frac{e}{{\eta }_{pc}}\frac{\phi {n}_{c}{f}_{c}}{{A}_{cc}}$, can be roughly estimated to 4 10^−5^ m.s^−1^ (40 *μ*m.s^−1^), by using typical numerical values: *e* ≈ 10^−6^ *m*,*η*_*pc*_ ≈ 510^−3^ *Pa*.*s*, *φ* = 0.1,*n*_*c*_ ≈ 160, *A*_*cc*_ ≈ 810^−11^ *m*^2^ where *f*_*c*_ has been estimated to 1 pN, as detailed in SI 2. This value is in the range on the ones experimentally observed in Fig. 5E. This simple phenomenological model yields numerical predictions in agreement with experiments and it captures the linear increase of the propulsive force with the distance to the domain center and the linear relationship between local force and mucus velocity. It shows that a ‘rigid-like’ motion at constant angular velocity is generated on the epithelial surface, enabling mucus to be transported without undergoing shearing forces.

## Discussion

To summarize the findings, we reported, on non fully differentiated and mature cultures, on the existence of epithelial domains where ciliary beats present a stable circular orientational order. These domains reveals circular flow patterns of mucus and of micron-size beads placed at the apical surface of the epithelium after mucus removal. This generic behavior was observed in human ALI cultures both commercial and made in-house from endobronchial biopsies on controls and asthmatic patients. The size of these domains, which ranges from tens of microns to centimeters, is controlled by the average ciliated cell density via a scaling law over more than three orders of magnitude. This physical quantity governs the distance over which the mucus is efficiently transported. Noteworthy, based on this curve, we found that the epithelium from severe asthma and COPD patients does not seem to be able to generate a sufficient number of active ciliated cells to ensure a long range transport of mucus. Our detailed quantitative analysis of 6 circular flow domains showed a radial increase in ciliated cell density and in orientation order of ciliary beats on these domains as well as a linear radial increase in mucus velocity. An important and notable finding is the radial regulation of both the ciliated cell density and the ciliary beat orientation which results in the linear radial increase of the net propulsive force. Finally, we developed a phenomenological model, which captures the observed linear relationship between the mucus velocity and the propulsive force, and predicts numerical values in agreement with the experiments. The *ν* and CBF values we found are respectively significantly lower and higher than those measured *in-vivo* in the trachea. On the basis of our findings, *ν* value of about 0.2 is enough to ensure a mucus transport over the whole culture chamber of centimeter size. A higher *ν* is therefore not required for efficiently transport the mucus on these ALI cultures. An increase in CBF could be a mechanism to provide the power necessary to handle a suitable mucus velocity. Indeed, the power provided by the ciliary beats is proportional to the total number of beats per unit time and thus, to both *ν* and CBF. A high frequency could therefore compensate a low ACDD to get a mucus velocity in the culture chamber in the range observed in the trachea. Such a phenomenon of high CBF compensating the lack of ciliary coordination is indeed physiologically relevant. It has been evoked for the clearance of amniotic fluid from newborn lungs^30^. Our study points out the fundamental role of the long-range spatial regulation of ciliated cell density and coordination of ciliary beats direction. Yet, the mechanisms underlying this regulation/coordination remain unclear. They are however crucial in chronic respiratory disease and also, in the development of the ciliated lung epithelium and in the regeneration of ciliary beat-induced flow after injury. Long-range fluid-structure hydrodynamic interactions combined with steric interactions are known to generate meso-scale coherent flow patterns, among which circular patterns, in active suspensions^31,32^. We suggest that hydrodynamic interactions in both periciliary and mucus layer may give rise to the emergence of circular flow patterns, driven by the circularly oriented drag force exerted by both the mucus and the periciliary fluid on ciliated cells. Ciliated cells could actively respond to this drag force, via a mechanotransduction mechanism, leading to spatial adaptation of ciliated cells density and of the orientation of ciliary beats. Although the mucus flow has been characterized in the present study, the flows in the periciliary layer, which may be paramount for mucus transport and ciliary activity are still poorly documented. Experimental and computational efforts on epithelial surface flows and collective motion in cilia assemblies are today crucial to shed light on the mechanisms governing the spatiotemporal organization of ciliary activity and to take the understanding of mucociliary clearance to the next level.

## Methods

### Endobronchial biopsy specimens and ALI cultures

Subjects were recruited for endobronchial biopsies at the Clinique des Bronches, de l’Allergie et du Sommeil(Asistance Publique HÃ´pitaux de Marseille, Marseille, France). The study protocol was approved by the local ethic committee and was part of the Bronchial Obstruction and Asthma Cohort (COBRA - Cohorte Obstruction Bronchique et Asthme) sponsored by the French National Institute of Health and Medical Research, INSERM [IDRGB 2008-A00284-51, Afssaps 2008-0113]. All subjects were informed about the nature and purpose of the study and provided written consent before enrolment. All experiments were performed in accordance with relevant guidelines and regulations.

*In-vitro* reconstituted bronchial epithelium were developed according to a previously existing protocol^17,33^ and briefly described in SI3. Alternatively, commercial ALI cultures (MucilAir) were bought from Epithelix.

### Cilia activity

Transmission light microscopy was performed on a Nikon Eclipse Ti microscope at 37 °C and 5% CO2 under a humid atmosphere. For each subject, three separate culture chambers were examined and on each culture chamber, the central zone and two opposite side zones, each one of area 255 *μ*m^2^ were observed. In each zone, the ciliary motion was recorded at 250 fps for 3 seconds with a camera Photron SA4. Then, using Fiji software, the average intensity of each pixel obtained from all images of the stack of 250 × 3 images was determined and subtracted, pixel per pixel to each image of the stack, in order to enhance the visualization of mobile cilia. In each zone, the number of ciliated cells with motile cilia was counted, the fraction of the surface area covered by beating cilia was measured, and the ciliary beating frequencies were determined over 20 cycles on 5 ciliated cells randomly chosen. Cilia located on the same ciliated cell were found to beat at the same frequency. *ν* and average ciliary beating frequency were determined for each patient after average of the measured quantities in the three zones and the three culture chambers.

### Measurement of transport properties

We imaged the mucus layer with a Photron SA4 camera or Andor Neo sCMOS camera (×10, ×20 and ×60 objectives) and computed the mucus velocity by tracking the motion of mucus heterogeneities or latex beads (microspheres, 1 *μ*m, red fluorescent) embedded in the mucus layer. The velocity and trajectory of latex beads on the epithelial surface after mucus removal were measured as follows. Mucus was removed by performing an apical washing with PBS supplemented with 10 mM DTT during 5 minutes, followed by 5 rinsings with PBS. A dilute suspension of latex beads in PBS was then placed on the epithelial surface in order to form a very thin layer (few microns) over the whole culture chamber surface. The motion of latex beads was then recorded with a Photron SA4 camera or Andor Neo sCMOS camera (×10, ×20 and ×60 objectives)

### Statistical analysis

In order to statistically analyze *ν* and CBF values, linear mixed-effects models were used. Dependent variables were *ν* and CBF, independent variable was the patient’s group (controls, mild asthma, severe asthma and COPD patients). A p-value < 0.05 is considered as significant. Statistical analyses were performed using R (version 3.1.3).

### Image processing

We developed a homemade routine in python to compute the CBF based on Fourier analysis. The orientation maps were computed by implementing in python the algorithm described in Püspöki *et al*.^34^ based on the structure tensor computation.

## Electronic supplementary material

Supplementary information

Beating cilia

Isolated ciliated cell(s)

Multiple swirls

Global rotation of beads on a mucus free epithelium

Chamber spanning swirl of mucus
