# Supplementary material for: Spatiotemporal organization of cilia drives multiscale mucus swirls in model human bronchial epithelium
Source: Sci Rep. 2018 Feb 5;8:2447. doi: 10.1038/s41598-018-20882-4 (PMC5799192; doi:10.1038/s41598-018-20882-4)
Supplement: Supplementary file 1 — Supplementary information [file 41598_2018_20882_MOESM1_ESM.pdf]

# Supplementary information for : Spatiotemporal organization of cilia drives multiscale mucus swirls in model human bronchial epithelium

**Mustapha-Kamel Khelloufi[1], Etienne Loiseau[1], Marc Jaeger[2], Nicolas Molinari[3,4], Pascal Chanez[5], Delphine Gras[5], and Annie Viallat[1,*]**

[1]Aix Marseille Université, CNRS, CINAM, Marseille, France
[2]Aix Marseille Université, CNRS, Centrale Marseille, M2P2 Marseille, France
[3]Institut Montpelliérain Alexander Grothendieck, CNRS, Univ. Montpellier
[4]Department of Statistics, University of Montpellier Hospitals, Montpellier, France
[5]Aix Marseille Université, CNRS, INSERM, LAI, Marseille, France
[*]viallat@cinam.univ-mrs.fr

## 1 Ciliary beat frequency

The average CBF measured on control subject is 18.3 Hz ($\pm$3.2). The average CBF does not significantly vary with the disease. It is found to be 16.6 Hz ($\pm$4.3) and 17.6 Hz ($\pm$4.2) for severe asthma and COPD respectively (see Fig. S 3). These frequencies are slightly higher than those reported in the literature either in-vivo, (12 to 15 Hz) (3)(26)(21), or in ALI cultures (12Hz)[?]. However, CBF is significantly more dispersed for patients with severe asthma (standard deviation of 4.2 Hz) than for control subjects (standard deviation of 3.2 Hz). After mucus removal, a small CBF increase to 19.7 Hz for controls, 17.2 Hz and 18.3 Hz for severe asthma and COPD respectively is observed. Interestingly, this small CBF increase is comparable for control subjects and for patients although it is believed that the rheological properties of mucus from severe asthma and COPD cultures differ from those of control ones. A typical spatial dispersion of CBF in a culture chamber is illustrated in Fig. 2B for a control subject (see Movie S1).

## 2 Orders of magnitude

In Eq. 2: $\frac{\varphi n_c f_c}{A_{cc}} v_r < sin\,\alpha >_r = \eta_{pc} \frac{(V_{SF}(r) + V_R)}{e}$.

This equation can rewritten and compared with the experimental observations of velocity versus $v_r < sin\,\alpha >_r$ variations:

$$V_{SF}(r) = \frac{e}{\eta_{pc}} \frac{\varphi n_c f_c}{A_{cc}} v_r < sin\,\alpha >_r - V_R \tag{1}$$

The magnitude of the force $f_c$ is that of the drag force exerted by the motion of a cilium tip moving at the relative velocity $V_{tip} - V_{SF}$ in the surface fluid, where $V_{tip}$ is the velocity of the cilium tip during the stroke. $V_{tip} = \frac{l}{\varphi T}$ with $l$ = distance traveled by the cilium tip in the surface fluid during the stroke (approximatively 5 $\mu$m) and $T$ the cilia beat period (about 0.07 s). Therefore, $V_{tip} \approx 700\,\mu m.s^{-1}$, much larger than the surface fluid velocity in the localized domains so that $V_{tip} - V_{SF} \approx V_{tip}$.

The force $f_c \approx \pi \eta_{SF} a V_{tip}$ , where $\eta_{SF}$ is the viscosity of the surface fluid $(10^{-3} Pa.s)$ and $a$ is the size of the tip $(5\,10^{-7} m)$, so that $f_c \approx 10^{-12}$N

1) The slope of the $V_{SF}$ versus $v_r < sin\,\alpha >_r$, $\frac{e}{\eta_{pc}} \frac{\varphi n_c f_c}{A_{cc}}$ , is roughly estimated $4\,10^{-5} m.s^{-1} (40 \mu m.s^{-1})$, by using typical numerical values: $e \approx 10^{-6} m, \eta_{pc} \approx 5\,10^{-3} Pa.s$, $\varphi = 0.1$, $n_c \approx 160, A_{cc} \approx 8\,10^{-11} m^2$. This value is in the range on the ones experimentally observed in Fig. 5A

2) The constant term $(v < sin\,\alpha >)_0 = \left( \frac{e}{\eta_{pc}} \frac{\varphi n_c f_c}{A_{cc}} \right)^{-1} V_R$ is slightly overestimated by the model. Indeed, the cilia

recovery velocity is of the order of $V_R = \frac{l_R}{(1-\varphi)T}$, where the distance $l_R$ traveled by a cilium during its recovery in the periciliary layer is of the order of 7 $\mu$m leading to $V_R \approx 100$ $\mu$m.$s^{-1}$ and $(\nu < sin\alpha >)_0 \approx 2.5$. The range of experimental values extrapolated from Fig. 4D is ten times lower, between 0.1 and 0.3. The frictional force is less than the one calculated by this crude model. A discrepancy was expected since we considered that the whole periciliary layer at the height of recovering cilia was flowing back at the cilia's velocity, even in empty zones. The real system is much more complicated with possible flows within an horizontal layer of periciliary layer and also vertically within the layer of thickness e. The small discrepancy observed between the model and experimental results is rather encouraging and might suggest that our model captured the main features of the transport.

## 3 Endobronchial biopsy specimens and ALI cultures

Briefly, fiberoptic bronchoscope was inserted by nasal way in trachea and biopsies were obtained using biospy forceps on a subsegmental bronchus of the left lower lobe. Bronchial epithelial biopsy tissue was dissociated and suspended in bronchial epithelial growth medium. After seeding in multiwell plates, cells were expanded in a flask and then plated on uncoated nucleopore membranes (24-mm diameter, 0.4-mm pore size, Transwell Clear; Costar, Cambridge, Mass) in a 1:1 mixture of bronchial epithelial growth medium and Dulbecco modified Eagle medium (Invitrogen, Carlsbad, Calif) to establish the air-liquid interface. Cells were maintained in culture for 28 days.

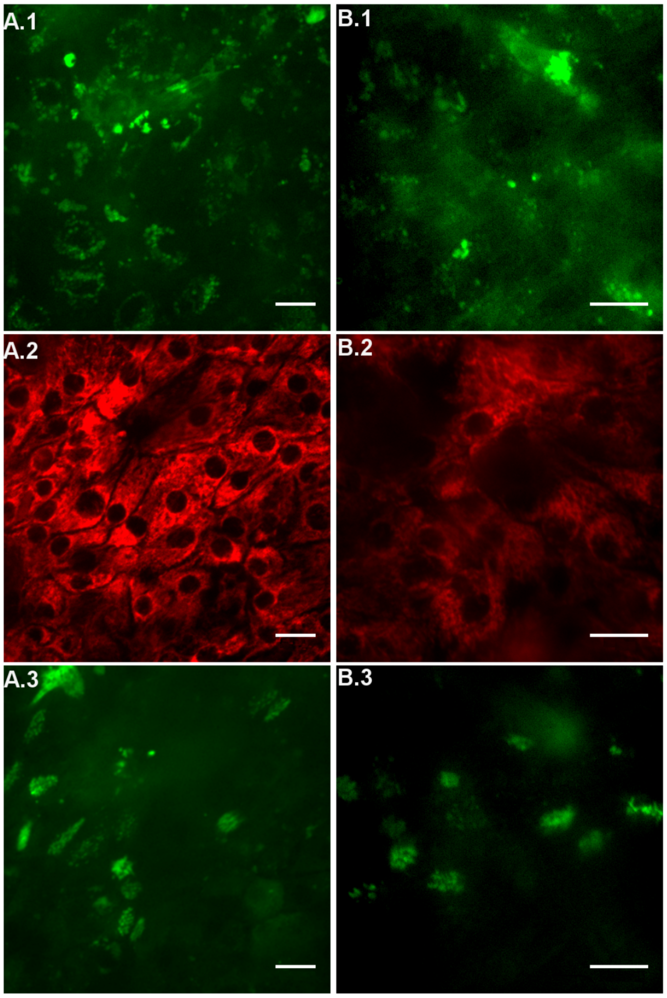


**Figure S1.** **Pseudo-stratified human ALI epithelium** - Confocal images of a control epithelium **(A1-A3)** and an epithelium with severe asthma which exhibits cohesion defects **(B1-B3)**. **(A2, B2)** Apical surface of epithelium (mitochondria specific staining in red). **(A3, B3)** Visualization of the cilia by tubulin specific staining (green) above the apical surface. Scale bars, 20 $\mu$m and the Z-step between successive slices is 7 $\mu$m.

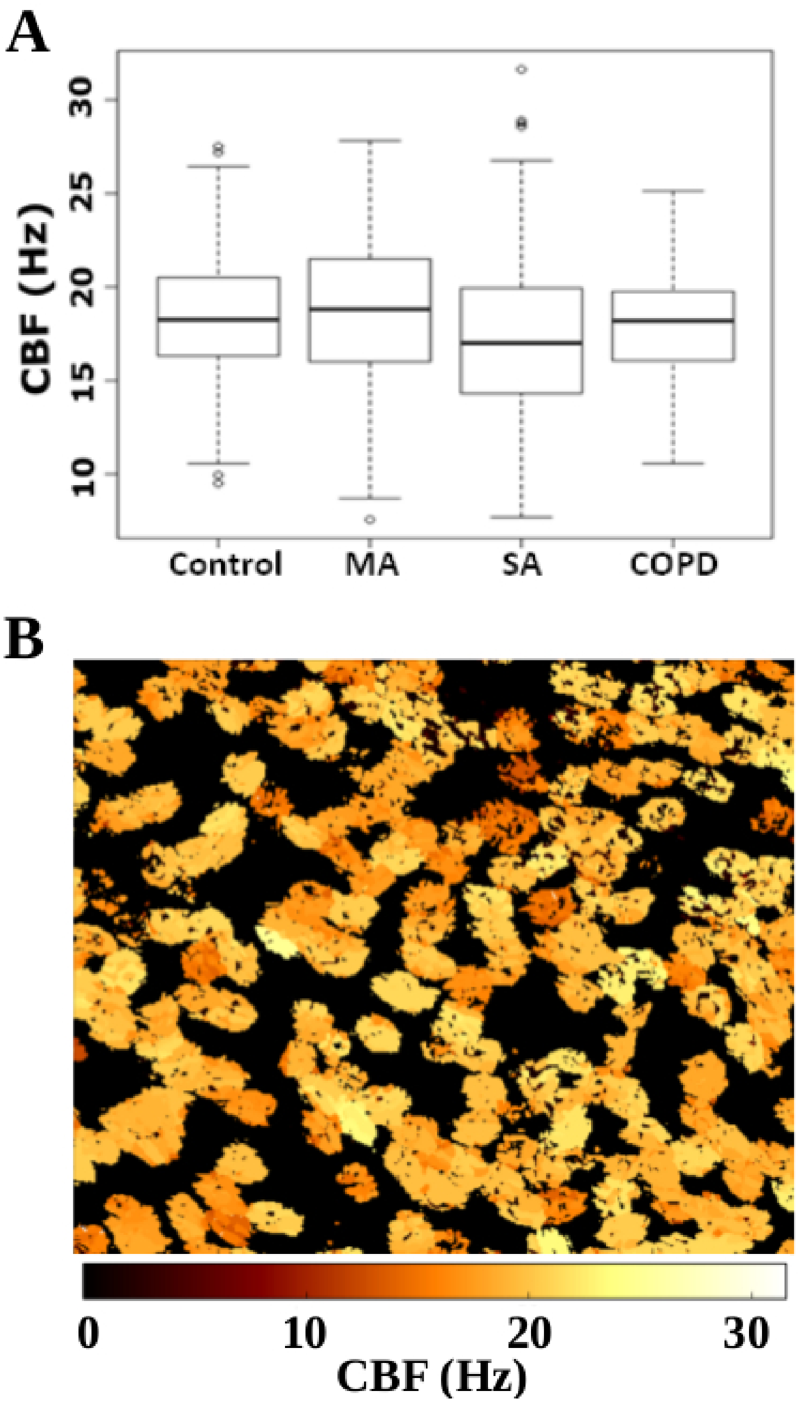


**Figure S2.** **Cilia beat frequencies (CBF) - (A)**The average CBF for control subjects is 18.3 Hz (± 3.2). It does not significantly vary with the disease: 18.1 Hz (± 4.4) for mild asthma, 16.6 Hz (± 4.3) for severe asthma and 17.6 Hz (± 4.2) for COPD. **(B)** Example of the dispersion of the ciliary beat frequency (CBF) for the culture shown in Figure 2C. The color codes for the frequency and the black areas correspond to zones without ciliated cells. Scale bar, 20 $\mu$m.

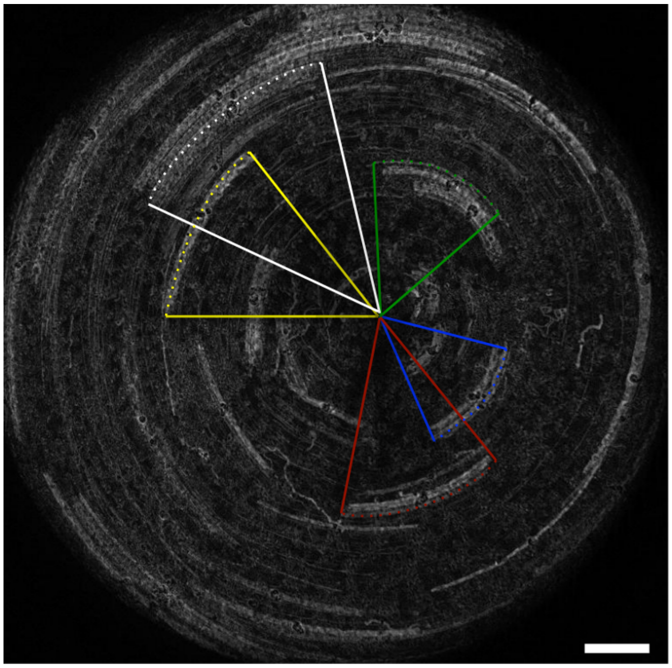

**Figure S3.** **swirls rotate at a constant angular velocity** - The image is a standard deviation projection. The trails results from the trajectories of dead cells/fragments embedded in the mucus. For a constant angle of 50 °, the curvilinear distance travelled by a dead cells/fragment increases linearly. Scale bar, 200 $\mu$m.

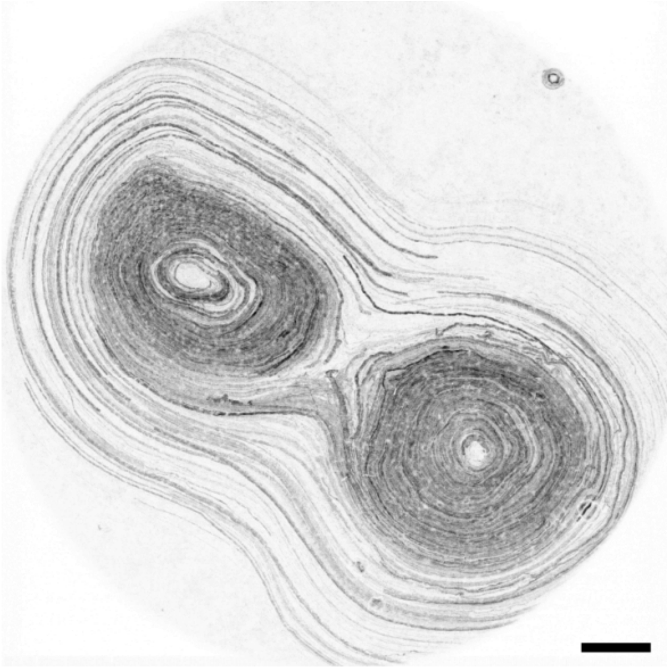

**Figure S4.** **Multiple swirls** - Two swirls close to each other. The background has been removed and the image results from a projection over 400 frames. Scale bar, 200$\mu$m

**Movie S1** **Beating cilia -** Beating cilia with a prefered orientation. The immobile background has been removed. The movie is slow down 5 times. Scale bar, 20$\mu$m.

**Movie S2** **Isolated ciliated cell(s) -** Beating cilia on ALI culture.

**Movie S3** **Multiple swirls** Two rotating swirls. The immobile background has been removed. Scale bar, 200$\mu$m

**Movie S4** **Global rotation of beads on a mucus free epithelium -** Rotation of micrometric beads over the whole culture chamber (fluorescence microscopy). The central part (2 mm diameter) of the chamber (6 mm) is shown. Scale bar, 200$\mu$m.

**Movie S5** **Chamber spanning swirl of mucus -** Rotation of mucus over the whole culture chamber. The central part (2 mm diameter) of the chamber (6 mm) is shown. Scale bar, 200$\mu$m.
